# Design and Modelling of Graphene-Based Flexible 5G Antenna for Next-Generation Wearable Head Imaging Systems

**DOI:** 10.3390/mi14030610

**Published:** 2023-03-06

**Authors:** Asad Riaz, Sagheer Khan, Tughrul Arslan

**Affiliations:** School of Engineering, The University of Edinburgh, Edinburgh EH9 3FF, UK

**Keywords:** fifth generation (5G), next generation mobile network (NGMN), long-term-evolution (LTE), core networks (CNs)

## Abstract

Arguably, 5G and next-generation technology with its key features (specifically, supporting high data rates and high mobility platforms) make it valuable for coping with the emerging needs of medical healthcare. A 5G-enabled portable device receives the sensitive detection signals from the head imaging system and transmits them over the 5G network for real-time monitoring, analysis, and storage purposes. In terms of material, graphene-based flexible electronics have become very popular for wearable and healthcare devices due to their exceptional mechanical strength, thermal stability, high electrical conductivity, and biocompatibility. A graphene-based flexible antenna for data communication from wearable head imaging devices over a 5G network was designed and modelled. The antenna operated at the 34.5 GHz range and was designed using an 18 µm thin graphene film for the conductive radiative patch and ground with electric conductivity of 3.5 × 10^5^ S/m. The radiative patch was designed in a fractal fashion to provide sufficient antenna flexibility for wearable uses. The patch was designed over a 1.5 mm thick flexible polyamide substrate that made the design suitable for wearable applications. This paper presented the 3D modelling and analysis of the 5G flexible antenna for communication in a digital care-home model. The analyses were carried out based on the antenna’s reflection coefficient, gain, radiation pattern, and power balance. The time-domain signal analysis was carried out between the two antennas to mimic real-time communication in wearable devices.

## 1. Introduction

In the modern era, communication technology has reached its peak, whereas the delivery of ultra-high peak data speeds, lower latency, enhanced network efficiency, high transmission reliability, data integrity, massive network capacity, users flexibility, and a more uniform availability to the users has brought a revolution in wireless technology [1,2]. This led to the tremendous innovation of 5G wireless technology which established an innovative unified network for universal connectivity of everyone and everything including people, machines, and devices. Thus, 5G is connecting the world in a universal Wi-Fi zone in the next decade. The user traffic is expected to burst to 10,000 times more compared to the current traffic, as millions of new devices will be connected using a 5G network. The United States (US) agency of the Federal Communications Commission (FCC) adopted frequencies ranging from 28 to 38 GHz for 5G standards that fell under the Ka-band, promising a lower rate of absorption, reduced path-loss, and minimal signal fading [3,4]. Similarly, in the U.K., the Office of Communications conducted 5G test beds on 26 GHz [5]. According to the next generation mobile network (NGMN) white paper [2], the latency in a two-way end-to-end (E2E) access is 10 ms in dense areas or 50 ms in areas influenced by environmental factors such as core networks (CNs), proxy servers, etc. The 5G deployment was started and based on two types, i.e., standalone and non-standalone. Most of the current deployments are non-standalone, which is the first stage of 5G deployment. At this stage, a new 5G network radio utilizes the existing 4G long-term-evolution (LTE) radio access and core networks (CNs) for mobility and coverage management of the connected devices [6].

## 2. Latest Healthcare Applications

In healthcare, 5G is mostly used for remote diagnosis, long-term monitoring, remote intervention, etc. There are numerous emerging applications of the 5G technology in healthcare’s robot-assisted remote surgeries, augmented reality (AR) assisted care services, hospital logistics automation, remote patient care, etc. [7]. Some of the up-to-date advancements are as follows.

- *Monitoring devices*: Most of the health-monitoring devices previously used 802.11 or 802.15.4 protocols which are currently part of the 5G technology. In [8], a detailed summary of commercially approved on-body and off-body medical devices for hospitals and medical clinics was presented. The use of medical sensors in the Internet of Things (IoT), specifically radio-frequency identification (RFID), and state-of-the-art technologies in IoTs were overviewed in [9].
- *eHealth Systems*: A 5G-based smart system was presented in [10], incorporating the integration of emerging technologies, e.g., wearable 2.0, machine learning, and big data algorithms for diabetes disease monitoring. A novel concept of the ‘m-health’ (a mobile small cell) was introduced in [11], where all the ultra-sound medical data were collected and processed, thereby called a smart ambulance. Wireless tele-surgery using video, audio, and remote service robots (RSR) was detailed in [12,13]. The authors in [14] presented a survey detailing the wearable antennas for 5G and their related future technologies. In a nutshell, further research is needed in this area for the proper utilization and implementation of 5G applications.
- *Robotics*: Robots are used extensively in healthcare, particularly in healthcare for elderly people. In [15], a prototype using a mobile robot and a computation unit was presented which communicated through ultra-reliable low latency communications (URLLC), a subset of 5G network architecture. An intriguing research was performed in [16], exploring the possibility of using a robot partner for healthcare; specifically, the people’s response and their attitude towards a companion robot were studied.
- *Virtual Reality (VR)/Augmented Reality (AR)*: Both VR/AR are good examples of the Tactile Internet, which is defined as a communication system with very low latency values. Both VR/AR require high computation capability, massive bandwidth, very low round-trip delay as well as high availability, data integrity, and security. The 5G network provides the necessary technology for implementing a highly reliable, low-latency VR/AR real-world experience that can be called haptic (stimulating the senses of touch and motion) in the real sense. In healthcare, specifically, remote surgery requires the performance of massive computation capability and readily available resources for real-time spontaneous service provision, which is fulfilled by 5G [17,18].
- *Healthcare Artificial Intelligence (AI)*: AI caused a revolution in data applications, as it made possible the processing and analysis of very complex algorithms. These advances in AI were made possible because of massive chunks of petabytes of data which were created by the networks and services available on the Internet. The driving applications of AI include; home care robots, medically assisted robotics arms and sensory devices, automation, and other intelligent mobile applications. The concept of intelligent Internet of Things (IIoT) was presented in [19], which connected the sensors and the cloud using 5G communication networks. The technologies related to IoT such as deep learning, big data mining, etc., are also described in this paper. Another study [20] presented the application of typical AI algorithms to 5G cellular networks. Additionally, ref. [21] presented eHealth support for a medical emergency. The paper described that the patient’s critical condition can be captured at the location of a medical emergency and necessary intervention can be made by incorporating mobile computing.

The above discussion provides a deep insight into the potential applications and advantages of 5G in healthcare. The usage of 5G in wireless communication, remote patient diagnosis, and continuous remote monitoring gained a growing interest in telemedicine [22,23,24]. The basic concept of telemedicine is that the patient’s biological parameters are monitored using multiple sensors. These sensors can be both on-body or implanted. The signals from these sensors are collected by receivers such as a mobile phone or a PC, whereas signals recorded are transmitted to diagnostic centres, doctors, or clinical facilities [25,26]. The idea of telemedicine is very beneficial as many lives can be saved by real-time monitoring and communication of sensitive patient data [27,28]. Several efforts were made in this area [29]. However, these solutions are still far from being adopted in routine medical investigations nowadays. The implementation technology still needs maturity, whereas many constraints that are limiting clinical implementation remain.

## 3. Flexible Electronics and Portable Systems

Flexible electronics generally refers to the class of electronic devices built on stretchable comfortable substrate materials, e.g., mostly plastics but also metal foils, flex glass, and paper as well. Flexible electronics received great attention in the past decade because of their potential to revolutionize human lives. Flexible electronics have whole application sectors including, telecom, solar cells, logic memories electronics, flexible sensors, displays, and medical devices. Apple inc. is on the verge of releasing some revolutionary wearable electronics, which will be more stylish, lightweight compatible, and mechanically durable including flexible wearable smart watches and smart bracelets [30]. Other such applications include the integration of ferroelectric oxide-based into flexible devices for very sensitive applications such as eyeglasses, 3D printing technology, and smart eardrop applications [31]. Similarly, daily devices such as cell phones, flexible smart watches, flexible smart bracelets, smart laptops, and computers need flexible wireless electronics. 

By 2023, it is estimated that the market share of flexible electronics will reach 40 billion [32,33]. Various flexible conductive materials are used for wearable applications purposes depending upon their dielectric properties, electrical conductivity, mechanical strength, accommodation to miniaturization, tolerance, etc. [34], including polymers, polyesters, and textiles, etc. [35,36]. Metal nanoparticles such as Ag (conductivity of 2.1 × 10^7^ S/m), and Cu (×10^6^ S/m) are used for designing flexible and stretchable electric circuits [37,38]. C additive polymers and their additives including polyaniline (PANI) [39], polypyrrole(PPy) [40], and C nanotubes [41] have been deployed with low to medium conductivity values. Graphene is becoming very popular for flexible electronics because of its extraordinary properties including exceptional mechanical strength, high thermal stability, high electrical conductivity, and biocompatibility. Graphene-based materials such as nano-flakes (6 × 10^5^ S/m) [42], graphene paper (4.2 × 10^5^ S/m) [43], and graphene-based fabric (2 × 10^5^ S/m) [44] have relatively good conductivity values and were, thus, used for flexible structure based antennas and wearable electronics.

Portable imaging systems are wearable anytime anywhere and are easy-to-move devices, which are contributing a great deal to the early detection and continuous monitoring of numerous fatal diseases such as strokes, heart diseases, neurodegenerative diseases, diabetes, arthritis, Alzheimer’s disease, etc. Currently used imaging systems widely used in medical healthcare such as CT scans, MRI, ultrasound, and X-rays are very bulky, costly, and not accessible in rural areas. These bulky systems are not movable to specific locations for diagnosis purposes [45]. Portable and wearable health-monitoring devices are the only solution to minimize the distance between the patient and the physician. These portable devices can be used for monitoring patients with critical diseases for long periods of time, and the data collected must be processed, stored and analyzed. 

Wearable devices used for medical diagnostics purposes need high bandwidths specifically for real-time patient monitoring. In [46,47], the authors detailed the possibility of adding some extra features to the 5G architecture in the future. These would enhance the communication bandwidth, solve addressing issues, and security improvement. The wireless integration of 5G into portable devices would allow the establishment of high-capacity, unified networks for versatile applications with the added advantages of device compactness, flexible structures, and resourceful fabrication [48]. It will increase the patient’s comfort through highly efficient and seamless collection and communication of the patient’s data [49]. Similarly, 5G technology helps in establishing a personalized database of individual patients based on personalized physiological indicators for the prevention and treatment of diseases. Nonetheless, the use of 5G makes imaging technology more sustainable by continuously collecting and analyzing the data and sharing it with the patient’s friends and family, doctors, etc. This keeps the patient self-motivated to implement the treatment in time while maintaining a good mood [50]. Thus, the 5G integration into medical diagnostic systems makes it more cost-effective in two ways, firstly by preventing the users from getting the disease in the early stage by continuous monitoring and secondly by providing out-of-hospital treatment, thereby reducing the on-the-spot treatment cost and long-term hospitalization cost [51]. 

The feasibility of using flexible electronics for a portable device is that these are based on organic and inorganic nanostructured materials [52], thereby providing the exact geometrical and performance features as required by a specific application [53]. Due to the nano-structure-based structure of the flexible materials, they can help design flexible devices on textile materials such as paper, silk plastic, etc. [54], with high mechanical flexibility and strength. An added advantage is that flexible materials are mostly inexpensive, specifically organic materials [55]. One such example is high-performance organic crystalline materials (OCMs) [56], which are widely used in the advanced electronics of the current era such as displays, image detection sensors, and flexible electronics-based artificial skin [57]. They not only provide good flexibility but also demonstrate excellent molecular diversity because of their nanoscale structures resulting in minimal gain defects required for smooth and uniform characteristics across the whole device structure [58]. The fabrication processes of flexible electronics, such as solution processing, inkjet printing, and even roll-to-roll, are inexpensive and easy to implement [59]. Flexible electronics have a low environmental impact and are biocompatible, especially organic flexible materials that are biodegradable and, thus, easily disposable after use [60]. 

In recent years, flexible antennas gained popularity for mm-wave based 5G architecture in different applications including cellular, vehicular, and wearable portable electronics, etc. [61]. There exists a shared interest among government departments, federal agencies, corporations, industries, and academia, in developing flexible antennae for deployment in extreme conditions. A few high-temperature applications include developing flexible antennas for monitoring the H2 safety in high-temperature gas-cooled reactors, development of communication solutions for non-line-of-sight (NLoS) communication in unmanned aircraft NASA, etc. 

A recent extensive study previewed the materials used for the flexible antennas, their fabrication methods and processes as well as their applications [62,63,64,65,66,67]. In [62], a practical report was presented in which the fabrication processes of both the textile and non-textile-based antennas were described. In [63,64], a comprehensive survey of different materials used for the fabrication of flexible antennas in the frequency range of mm-wave to very high frequencies was presented. In [65], the flexible antennas were covered in detail, focusing on the materials and fabrication techniques along with specific applications and limitations. The wearable flexible antennas operating in ultra-wide band (UWB) for frequencies ranging from 3 GHz to 10 GHz were detailed in [66] along with their applications in the wireless body area network (WBAN) systems. A survey on the various types of implantable antennas, their specific design requirements for specific applications, and performance analysis of different implantable antennas was presented in [67]. The choice of flexible wearable antennae for wireless applications depends mainly upon the channel characteristics of the wireless environment, the transmission capability of the channels as well as the operating frequency [68]. In addition, the major factors defining the antenna performance include the type of flexible material used, the fabrication technique as well as the electrical, and mechanical properties, and the physical geometry of the antenna. The substrate material for the antenna is chosen based on the material’s minimal dielectric loss, low relative permittivity, and low value of the thermal coefficient of expansion as well as high thermal conductivity [69]. A tradeoff between the antenna size and increased efficiency is taken for meeting these constraints. There are three types of substrates used for the fabrication of flexible antennas including thin glass, polymers, and metal foils [70]. Polymers or plastic-based materials are becoming very popular for flexible antennas because of their robustness, flexibility, wettability, and stretchability. One such example is Kapton polyimide with a dielectric constant of 2.91, a loss tangent of 0.005, and high transition temperature (Tg), which is one of the most preferred substrate materials, for flexible antennas in the previous literature [71,72,73,74,75]. 

The authors in [76] reported a flexible, washable fully textile-based antenna which was wearable and reusable operating at frequency bands between from 3 to 20 GHz. The authors had undertaken the design and analysis of the antenna for smart garments and wearable medical monitoring purposes in detail. For instance, flexible graphene-based antennas and arrays were designed using the flexible polyimide substrate with a wide bandwidth, operating at 15 GHZ [77]. This antenna supported high-speed transmission; however, the bandwidth was limited and not sufficient for large data applications such as portable health monitoring systems in telemedicine. This problem can be solved by designing a wearable flexible antenna for 5G frequency ranges between 26 and 60 GHz. In another study, a flexible wideband slotted monopole antenna was designed for a millimeter wave range [78]. The antenna exhibited an ultra-high bandwidth of 26 GHz, i.e., from 18 to 44 GHz; however, the radiation efficiency was limiting at 55% and the gain maximum value was 1.45 dBi. Similarly, in [79], a comparison was made between CPW-fed antennas upon PET and Epson paper at 20 GHz. In [80], the modelling of flexible coplanar waveguide fed (CPW) antennas over the 5G range of 23 to 29.5 GHz was performed. The substrate material utilized was transparent PET. An LCP substrate-based proximity antenna for the 24 GHz range was designed using inkjet-printing technology [81]. These antennas operated at different frequencies owing to the fact that these were reconfigurable antennas. Another reconfigurable wearable antenna operating at 20.7–36 GHz was designed using inkjet printing [82]. This antenna incorporated various switch configurations for operation purpose. 

A flexible micromachined patch antenna operating at 60 GHz was designed using the PDMS substrate for assessing this technology with other market-available technologies [83]. A high-gain flexible mm-wave antenna was designed with a peak gain of 11.35 dBi at 35 GHz [84]. This antenna was unique in a way as it maintained a consistently high gain of above 9 dBi over the complete Ka-band. Another study proposed the design and analysis of an electromagnetic bandgap (EBG) based mm-wave MIMO antenna at 24 GHz [85]. The authors in this paper presented the design of a flexible Rogers-based substrate for on-body wearable applications with bending ability. 

Plenty of research was published on flexible antennas over UWB and lower frequency ranges, whereas a small amount of literature was present on flexible antennas at mm-wave spectrum for integration of 5G and beyond technologies for the healthcare portable wearable systems [86]. Thus, a need for a high bandwidth antenna arises that could be used for 5G front ends in telemedicine systems, which communicate continuous and real-time monitoring data over the 5G frequency spectrum with good efficiency and gain values. The antenna should be flexible enough to be integrated into flexible wearable devices. In order to minimize the attenuations of the antenna, gain should be optimized in comparison to the bandwidth due to the gain-bandwidth tradeoff. 

The motivation behind this paper is to design a novel flexible antenna for 5G communication in Ka-band, for telemedicine purposes. This antenna will enable the wearable portable device to perform the real-time monitoring of the patient and communicate the collected data over the 5G frequency band to the desired location for remote analysis purposes. The antenna has a graphene-based radiating patch on a Kapton polyimide substrate that makes it flexible and wearable. The antenna is simulated and analyzed for individual performance in free space, and the time domain signal analysis for the front-to-front antenna’s communication. The antennas were analyzed for their performance and their ability to operate in a stationary care-home model for telemedicine purposes. The analysis was carried out in the presence of a human model in the home-care scenario by keeping the antenna in line-of-sight (LOS) and non-line-of-sight (NLOS) at variable distances, positions, and locations from each other.

## 4. Antenna Design

**Figure 1:**
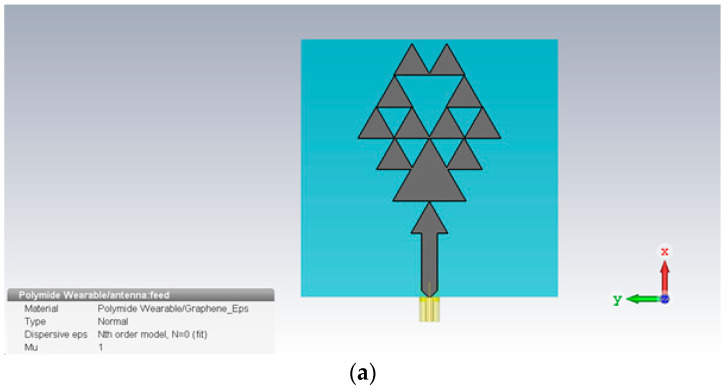

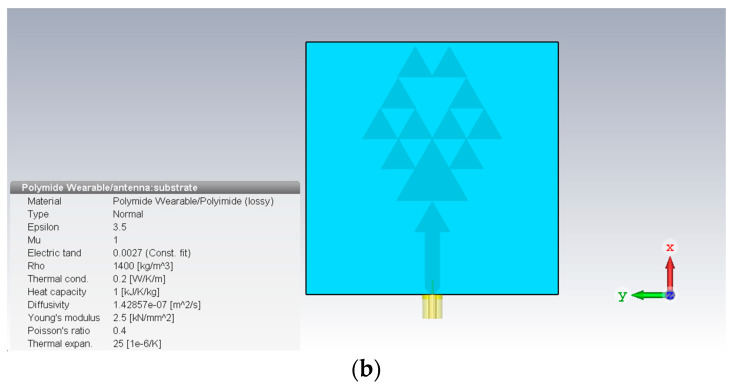
(**a**) Front view of the proposed antenna with Antenna Patch (**b**) Antenna Substrate.

The antenna design was carried out using the CST Microwave Studio software. Flexible substrate Kepton HN polyamide with a thickness of 1.5 mm was used to make the structure flexible and strong with a dielectric strength rating of 7700 V/mil for a 0.001 thick film and a tensile strength of 221 MPa. The antenna was fed using coaxial feeding, i.e., the inner conductor of the coaxial cable was soldered into the radiating patch, extending through the dielectric, while the outer conductor was attached to the ground. This technique helped in matching the cable impedance with the input impedance of the antenna by placing the feed at any suitable location on the patch. This technique was easy to fabricate with minimum spurious radiation. Its major aim was to enhance the antenna gain, narrow bandwidth, and impedance matching [87]. The central strip of the coaxial cable had a 4.88 mm width which was equivalent to the 50 Ω impedance line for this material. The patch was designed using a fractal structure that increased the conductive path without affecting the antenna’s radiation properties. The antenna front view is shown in Figure 1 below. The antenna was made of an 18 µm thin graphene film for the conductive patch and ground with electric conductivity of 3.5 × 10^5^ S/m, as shown in Figure 1a. The radiative patch was designed in a fractal geometry that brings many benefits over a plane structure. 

The fractal patch made the design operate consistently over a wideband with a bandwidth of 14 GHz with faster communication. The fractal design allowed instantaneous spectrum access, which meant a single antenna could be used instead of many. The fractal structure survived in the harshest conditions which made it very useful for frequently used flexible wearable portable devices. The substrate was designed over a 1.5 mm thick Kapton-based flexible polyamide material with a permittivity of 3.5 F/m, as shown in Figure 1b. 

The design dimensions are summarized in Table 1 in detail. The width of the feedline was kept at 4.88 mm to obtain an optimal value of radiation efficiency (62%) and gain (9.1 dB at 33 GHz) which decreased as the width was increased. A tradeoff was made between the height of the substrate (1.575 mm) and a minimum graphene patch height (0.018 mm) which helped in increasing the radiation efficiency as well as maximizing the antenna’s bandwidth at around 14 GHz. The patch fractal geometry consisted of a larger triangle and ten smaller triangles with side lengths in a ratio of 1 to ½, i.e., 6.6 mm to 3.3 mm designed in a symmetric combination to provide radiation efficiency and structural flexibility. 

## 5. Simulation Results

In this section, the simulation results of the proposed antenna are presented in detail. CST Microwave Studio by Dassault Systèmes’ was used for our design which was based on the finite integration technique (FIT) in the time domain. The graphene-based patch elements were embedded into the fractal geometry to maintain optimal antenna performance. The antenna geometry was optimized for analysis in terms of return loss, voltage standing wave ratio (VSWR), and gain of the antenna. The key performance parameters of the design were described as follows. 

### 5.1. Return Loss (S_11_) and Voltage Standing Wave Ratio (VSWR)

**Figure 2:**
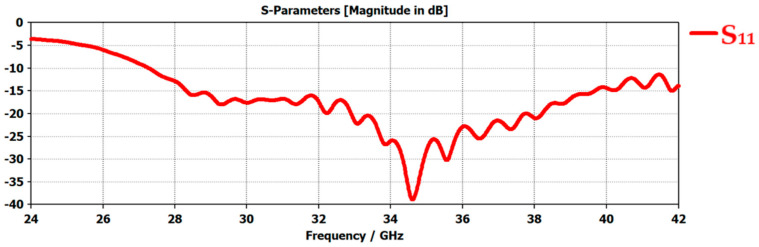
S-parameter, S_11_, of 5G Flexible antenna in free space.

Figure 2 shows the return loss of the antenna which is represented by the scattering parameters (S-parameters) graph. When the antenna was energized, some of the antenna power was transmitted while another part of the input waves was reflected or lost in the environment. The value of return loss shows the number of reflected waves in the antenna structure. This reflection occurred due to many factors both inside the antenna structure and because of environmental factors. The two main reasons that caused the return loss to occur include (a) discontinuities at connections and (b) impedance mismatches. The value of the return loss of the antenna should be less than −10 dB for proper operation. This is because at this value the antenna’s VSWR will be >2 which means that around 30% of the transmitted power will be reflected, whereas 70% of the input power will be transmitted successfully. The simulated return loss of the proposed antenna had the minimum value at −38.9 dB at 34.6 GHz resonant frequency, as shown in Figure 2. The antenna had a wide operating bandwidth of around 14 GHz that ranged from 27.3 GHz to around 41.5 GHz, with S_11_ below −10 dB. The practical influence of the return loss on telemedicine is that lower the value of return loss, higher will be the power transmission towards the receiving end in the telemedicine system. 

### 5.2. Voltage Standing Wave Ratio (VSWR)

**Figure 3:**
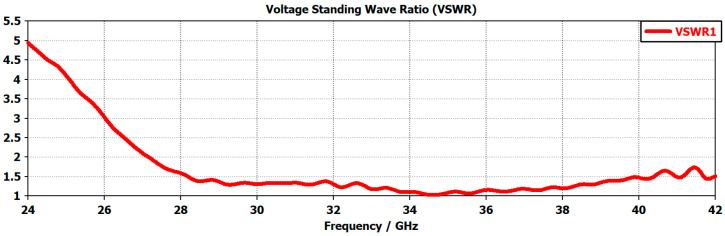
Antenna’s VSWR versus frequency response.

VSWR is a measure of the amount of mismatch between the antenna and the feedline connecting to it. The smaller the value of VSWR, the better the antenna is matched the transmission line and more power is transmitted. For an antenna to operate with a good power transfer value, the antenna’s VSWR should be <2 [88]. The graph in Figure 3 shows the value of VSWR of the antenna with values < 2 for the frequency range of 27.2 GHz < f < 41.5 GHz, which confirmed that the proposed antenna will operate efficiently between the range of 27.3 GHz and around 41.5 GHz. 

### 5.3. The Practical Influence on the Telemedicine System

The practical influence of the return loss and VSWR on telemedicine is that the lower the value of return loss and VSWR, the higher the power transmission towards the receiving end in the telemedicine system will be. In this design, the return loss value of −38.9 dB and a VSWR of 1.02 at 34.6 GHz resonant frequency results in a small value of Reflection co-efficient (Γ) of 0.011. This shows that a good amount of power was transmitted towards the receiving end. Similarly, the reflected power was only a minor percentage, i.e., 0.013%, with a high transmitted power of 99.987%. In a nutshell, the overall mismatch of the antenna was a minute value of 0.0056 dB. 

### 5.4. Radiation Pattern, Directivity, and Gain

**Figure 4:**
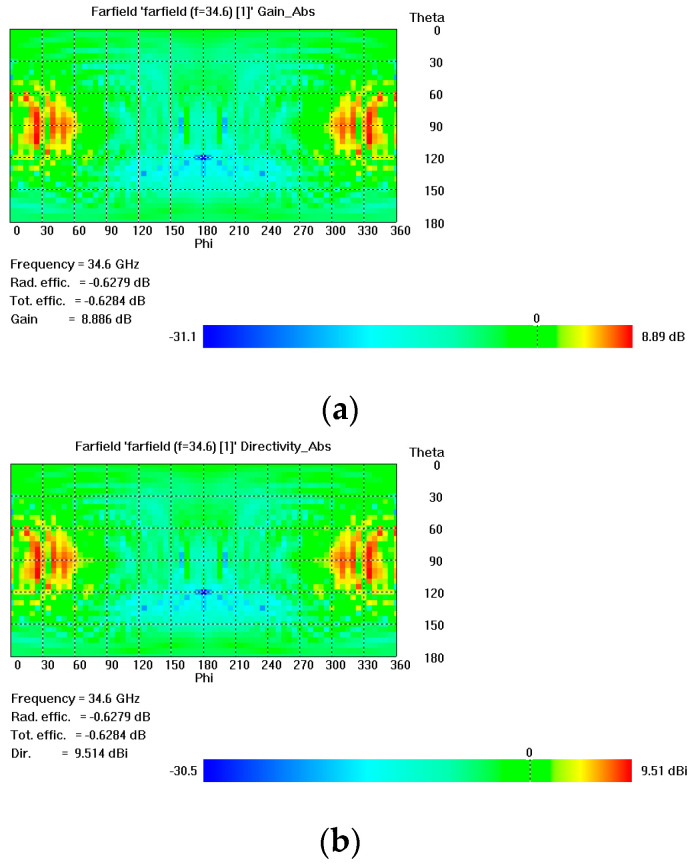
2D plots representing (**a**) gain of the antenna (**b**) directivity of the antenna.

The gain of an antenna represents its ability to radiate less or more in any direction compared to the hypothetical antenna. For example, if, in theory, an antenna can be made perfectly spherical, it would radiate equally in all directions. The higher the gain of the antenna, the better the antenna’s ability to transmit in a particular direction. Similarly, the directivity of an antenna shows the power density of an antenna in the maximum radiation direction concerning the average power density of the antenna in all directions. The higher the antenna’s directivity value, the better the radiation concentration in a particular direction and, therefore, the farther the radiation beam will travel. Figure 4 shows the 2D pattern representing the directivity and gain of the flexible antenna. Figure 4a shows that the maximum gain of the radiator increased from 27 GHz to 41 GHz with the highest value of 8.89 dB at 33 GHz. Figure 4b shows the maximum value of directivity at 9.51 dB.

## 6. Time Domain Communication Analysis

**Figure 5:**
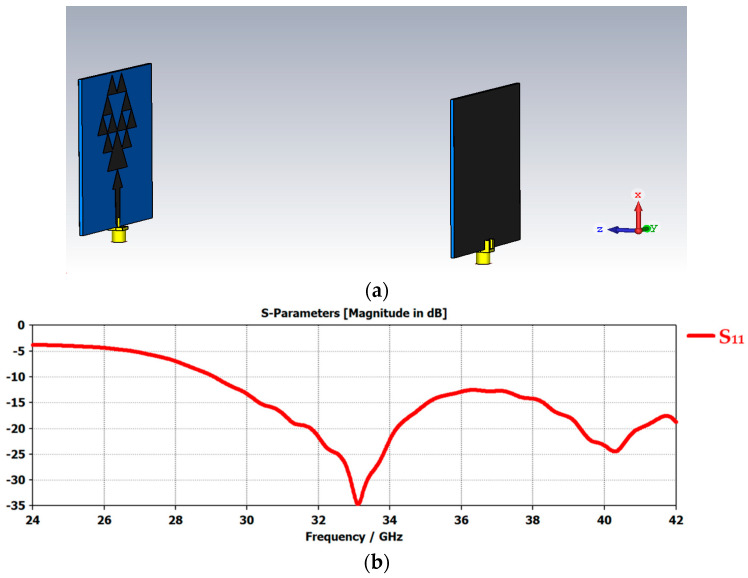
(**a**) Time domain analysis of front-to-front antennas in free space. (**b**) S_11_ graph of the time domain antenna communication in free space.

**Figure 6:**
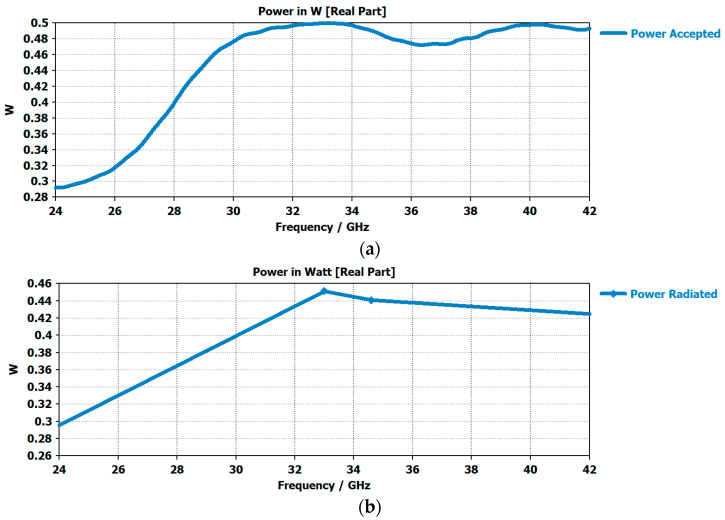
(**a**) Plot of power accepted by the antennas in the front-to-front communication in free space (**b**) The graph showing the amount of radiated power towards the receiving antenna.

A front-to-front antenna model is presented in Figure 5a, in which the two prototypes of the flexible 5G antennas were simulated and the time domain communications were analyzed. The return loss value of −35 dB shows that there was a good amount of power transfer between the antennas, as shown in Figure 5b. The communication bandwidth was a wide bandwidth of around 7 GHz ranging from 29 GHz to 36 GHz. This made our design a good candidate for use in telemedicine, which is simulated in the form of a care-home model in the next section. The graph comparison of various input and output signals is shown in Figure 6a,b as follows.

## 7. Care-Home-Model in CST for Telemedicine Simulation

Today’s era can truly be called the era of data-centric computing with petabytes of data generated every day. The Internet of Things (IoT) caused a revolution in smart-health-care research by connecting humans and devices [89]. The authors in [90] proposed a new name for this research area: the Internet of Health Things (IoHT). Innovative data processing models and tools are the need of the day for the collection, storage, and processing of the increased amount of data from IoTs and information sources. Big data analytics (BDA), on the other hand, has left both the public and private healthcare sectors with a massive amount of data that they never had access to before [91]. AI integration into the healthcare sector is increasing gradually in order to cope with the global challenges in the healthcare sector including the increasing ageing population [92], shortage of medical healthcare staff [93], and soaring costs [94]. Patients suffering from chronic diseases such as heart diseases, neurodegenerative diseases, diabetes, arthritis, Alzheimer’s disease, brain and heart strokes, Parkinson’s disease, etc., need early detection and continuous monitoring. The currently available detection and monitoring devices are not only static and bulky but costly as well. Therefore, portable and flexible wearable healthcare devices equipped with 5G communication technology are the need of the day. 

**Figure 7:**
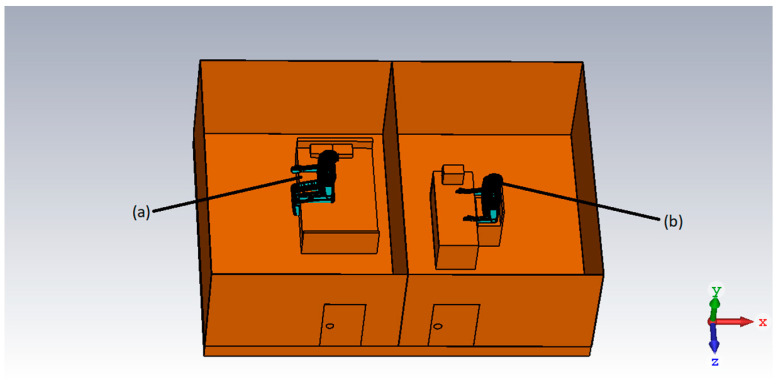
Digital model of the home care with the human model (a) Human model (patient) with the antenna mounted in the wearable head device (b) Human model (doctor) at the receiving end.

CST Microwave studio was used to design a digital model for a home-care scenario with the flexible 5G antenna placed in the two different rooms. Figure 7 shows that Antenna.1 was placed in a portable wearable device on the patient’s head, whereas antenna.2 was placed at the receiving end in a doctor’s room with a brick wall separation between the two antennas. The digital model was created in SolidWorks (properties of each item in the model were assigned in SolidWorks), and imported into the CST studio for simulation purposes. The human/patient model carried the properties of the human skin available in CST studio, the table at the receiving end was made of wood, and the wall was made of bricks all with different dielectric properties to imitate a real-case scenario. 

**Figure 8:**
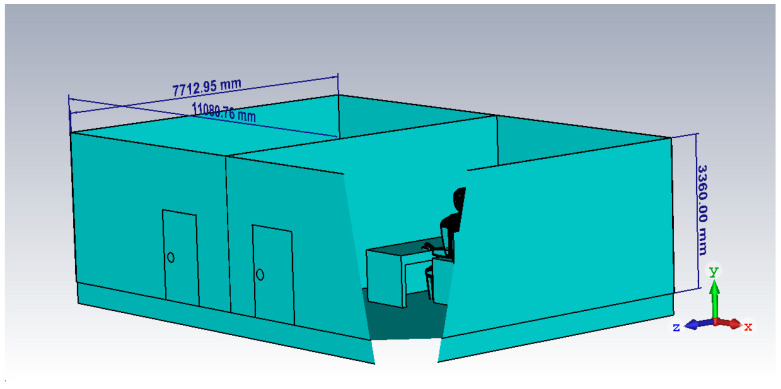
VHCM Dimensions.

The dimensions of VHCM are summarized in Table 2 and in Figure 8 as follows. The room dimensions were kept at 7.7 m × 5.5 m × 3.3 m. The human model height was taken as 1.6 m, i.e., an average height of a normal human being. The two antennas were kept 3 m apart from each other in separate rooms mimicking both the LoS and NLoS scenarios. The table size was taken as 0.75 m × 1.5 m. The thermal resistance (R) of the brick wall used was 2.63 and the thermal transmittance (U) was 0.38. The permittivity of the room wall was 3.56, whereas the permeability of the brick wall was 0.79%. 

**Figure 9:**
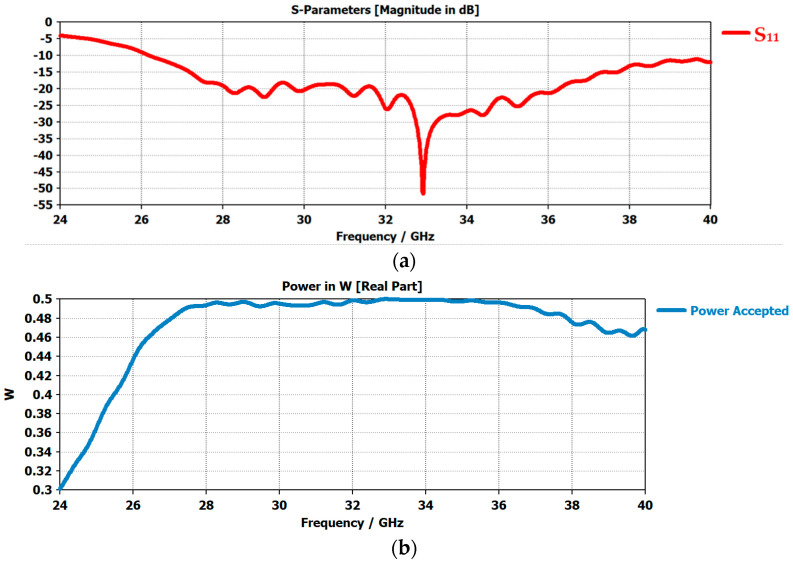
Antennas simulation in the front-to-front line-of-sight (LOS) communication in digital home care model (**a**) S_11_ versus frequency plot of the Los communication between the antennas (**b**) Power accepted by the receiving antenna.

The value of return loss obtained after the simulation not only depended upon the dielectric properties of the objects present but also upon the position of the patient and antennas both at the transmitting and receiving end. Figure 9 shows the return loss and power values when the two antennas were facing front-to-front in line-of-sight (LoS) in the home care model. Figure 9a shows a good power transfer with the S_11_ of −51.4 dB, whereas Figure 9b shows that the power transfer between the antennas shoots to a maximum after 27 GHz of frequency and stays at its maximum value till 42 GHz. Both of these graph depict that a good value of power was transmitted from the transmitter antenna to the receiver antenna in the line-of-sight (LOS) communication in the virtual home care model (VHCM). 

**Figure 10:**
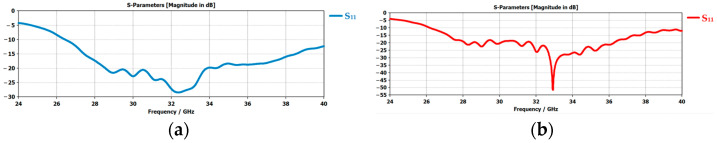
Comparative analysis of the S_11_ versus frequency plots (**a**) S_11_ versus frequency plot antennas simulation in the front-to-front no-line-of-sight (NLoS) communication in digital home care model (**b**) S_11_ versus frequency plot of the LoS communication between the antennas.

A comparative analysis of the return loss of the LOS versus NLOS communication was performed in Figure 10. Figure 10a shows that the S_11_ of the antennas placed in the NLOS was almost the same value of −55.5 dB as compared to the S_11_ of the antennas in the LOS situa, as shown in Figure 10b. Thus, the simulation shows that the proposed antennas performed equally well when they were simulated in different scenarios of LOS and NLOS in the VHCM. 

## 8. Conclusions

The fusion of 5G into healthcare systems has a huge implementation potential both for the public and private healthcare sectors. Unobtrusive mm-wave communication is an effective method for collecting and transmitting useful data from healthcare devices. This research focused on the design and integration of flexible 5G antennae for wearable portable devices used for healthcare purposes. Firstly, the time domain signal communication of the two front-to-front antennas in free space was analyzed and optimized. Secondly, the antennas were analyzed for their performance and their ability to operate in a stationary care-home model for telemedicine purposes. The analysis was carried out in the presence of a human model in the home-care scenario, by keeping the antenna in line-of-sight (LOS) and no-line-of-sight (NLOS) at variable distances, positions, and locations from each other. The analysis showed that data transmission between the transmitting and receiving antennas was varied by the antenna’s position, location, and distance. The value of return loss remained almost the same in both the LOS and NLOS scenarios. However, the power transmitted was maximum in the LOS scenario with a minimum distance (in our case) in between the two antennas. Future work should aim to fabricate the 5G flexible antenna and verify the results in a real-time scenario. Additionally, the fabricated antenna can be implanted in a portable wearable device and the actual scenarios of data collection and transmission should be performed by keeping the antennas at different positions, heights, and distances. 

## Figures and Tables

**Table 1:** Design parameters of 5G flexible antenna and their values.

Antenna Parameters	Label	Value (mm)
Small Triangle Side Length	D_f_	3.3
Width of the Feedline	W_f_	4.88
Height of Graphene Patch	H_g_	0.018
Height of the substrate	H_s_	1.575
Side Length of the bigger Triangle	D_T_	6.6
Patch Length	L_p_	40
Patch Width	W_p_	80
Length of the Feedline	L_f_	20

**Table 2:** Virtual Home Care Model Dimensions.

Dimensions	Values (meter)
Room Length (meters)	7.7
Room Width	5.5
Room Height	3.36
Human Model Height	1.6

## Data Availability

Not applicable.
